# Ciabatta Bread Incorporating Goji (*Lycium barbarum* L.): A New Potential Functional Product with Impact on Human Health

**DOI:** 10.3390/foods12030566

**Published:** 2023-01-27

**Authors:** Vincenzo Sicari, Rosa Romeo, Antonio Mincione, Simone Santacaterina, Rosa Tundis, Monica Rosa Loizzo

**Affiliations:** 1 Department of Agraria, Mediterranea University of Reggio Calabria, 89122 Reggio Calabria, Italy; 2 Department of Pharmacy, Health and Nutritional Sciences, University of Calabria, 87036 Rende, Italy

**Keywords:** *Lycium barbarum*, phytochemical content, antioxidant activity, lipase inhibition, α-amylase inhibition, α-glucosidase inhibition, consumer’s acceptance

## Abstract

This work investigated the phytochemical content and bioactivity of *Lycium barbarum* collected in Calabria and evaluated, for the first time, the possibility of enriching traditional ciabatta bread with goji fresh flesh puree. For this purpose, goji flesh puree, bread, and bread enriched with 20% and 40% goji flesh puree (G20 and G40 samples, respectively) were subjected to several analyses. Selected compounds were quantified by UHPLC analysis in both goji fresh flesh puree and after simulation of the cooking process. The impact of the addition on key enzymes (lipase, α-amylase, and α-glucosidase) related to metabolic syndrome was assessed together with the antioxidant properties. Texture, colourimetric, and sensory analyses on enriched bread were performed to evaluate consumer acceptance. Despite cooking, the enriched bread maintained good levels of bioactive compounds compared to the berry pulp alone (*p* < 0.01). The enriched bread showed the ability to protect against lipid peroxidation, with IC_50_ values of 6.88 and 6.52 μg/mL for samples G20 and G40, respectively, after incubation for 30 min (*p* < 0.01). Although less active than the control, the enriched bread showed inhibitory activities against the enzymes involved in the digestion of carbohydrates. From a sensory point of view, the addition of goji fresh pulp puree slightly modified the appearance but not the flavour and taste of the bread. Collectively, our results support the potential healthy function of this baked product.

## 1. Introduction

Several epidemiological studies have highlighted the relationship between diet and non-communicable diseases (NCDs) [1]. NCDs have emerged in several countries as the leading cause of morbidity and mortality. The major NCDs related to diet are diabetes mellitus and cardiovascular diseases. The key diet-related risk factors for these NCDs include high blood lipids, hyperglycaemia, overweight/obesity, and hypertension. A healthy diet can affect not only short-term health but also long-term health and can help reduce risk for many diseases [2].

Since ancient times *Lycium barbarum* L. (goji) berries, roots, and leaves have been used as food in raw, dried, and processed forms as well as a traditional herbal remedy in Central Asia [3,4]. This perennial deciduous shrub is characterised by ellipsoid orange-red berries and a sweet-tangy flavour [4]. In recent years, the consumption of goji berries, designated as “superfruits,” and goji-based products has increased globally thanks to the identification of secondary metabolites that exert health benefits, mainly including polyphenols, carotenoids, alkaloids, and polysaccharide complexes. Several studies have shown the hypoglycaemic, hepatoprotective, hypotensive, and antipyretic activities of *L. barbarum* [4,5].

Moreover, polysaccharide extracted from *L. barbarum* is found to have hypoglycaemic, anticancer, immunological, and antioxidant properties [5,6].

The European Commission has declared the consumption of *L. barbarum* berries as safe. Furthermore, in Italy, legislative decree n. 169 on 21 May 2004 by the Ministry of Health included goji berries in the list of plant extracts that can be used as food supplements with antioxidant activity. As a consequence, food researchers and the industry have been working hard to design and develop foods with extra health benefits for the consumer, especially if formulated with the addition of natural products [7].

Currently, there are numerous products formulated with goji berries on the market, including beverages, jam, ice cream, meat products, yoghurt, and cheese, as well as bakery and confectionery products, etc. [8].

Among functional foods, the category of functional baked products has received increasing attention during the last ten years. These products are indeed foods naturally rich in macronutrients, such as starch and dietary fibre, and can be easily enriched with micronutrients, such as antioxidants and minerals. Their transport and storage are less demanding than other categories of functional foods (e.g., yoghurt) and, more importantly, their daily consumption makes them particularly attractive for carrying bioactive substances capable of promoting beneficial effects on human health [9].

Oxidative stress plays a key role in the pathogenesis of several health problems, including metabolic syndrome [10]. This condition refers to a cluster of common abnormalities, including abdominal obesity, elevated triglycerides, reduced high-density lipoprotein (HDL)-cholesterol levels, and impaired glucose tolerance, with a prevalence estimated at more than 30% and 24.3% in the United States and Europe, respectively [11,12].

In fact, Găman et al. [13] demonstrated that in patients with DMT2, oxidative stress impairs glucose uptake in tissues and reduces insulin secretion by pancreatic β cells.

Natural products have attracted much interest as a potential approach to prevent and treat metabolic syndrome [14]. Several possible strategies have been normally applied to counteract metabolic syndrome and obesity, especially in the initial and preventive phases of the disease. Among them, the inhibition of α-amylase and α-glucosidase, the enzymes responsible for carbohydrate digestion, is one of the most adopted intervention strategies to reduce the post-prandial glycaemic peak [15]. Additionally, the inhibition of lipase, which is involved in the breakdown of triglycerides into absorbable free fatty acids, has a subsequent hypolipidaemic effect [16].

This work aimed to investigate the phytochemical content and bioactivity of *Lycium barbarum* collected in Calabria and evaluate, for the first time, the possibility of enriching traditional “ciabatta” bread with goji fresh flesh puree. Consuming this new functional food product, with a strong relationship with the territory, could help to prevent metabolic syndrome and associated pathologies due to its antioxidant properties and inhibitory activities against key enzymes, such as α-amylase, α-glucosidase, and pancreatic lipase. Moreover, despite scientific evidence of the beneficial effects of goji berries, data on bread enriched with goji are not available in the literature. The choice of ciabatta bread as a possible matrix is linked to the fact that this bread is widely consumed throughout Italy and is recognisable by large cavities in the crumb and a brown, crunchy crust.

For this purpose, goji fresh flesh puree, goji flesh puree after cooking treatment, bread, and bread enriched with 20% and 40% goji fresh flesh puree were screened for their phytochemical content and potential bioactivity related to metabolic syndrome and obesity. Levels of total polyphenols, flavonoids, carotenoids, and anthocyanins were examined. The in vitro antioxidant activity was assessed using a multi-target approach (ABTS, DPPH, β-carotene bleaching, and FRAP assays). The α-amylase, α-glucosidase, and lipase inhibitory activities were also assessed. To evaluate consumer acceptance, colour, texture, and sensory analyses were performed. PCA analysis was used to correlate the bioactivity to goji phytochemical content.

## 2. Materials and Methods

### 2.1. Chemicals and Reagents

Chemicals and reagents were obtained from VWR International (Milan, Italy) and Sigma-Aldrich Chemical Co., Ltd. (Milan, Italy). Lycopene and β-carotene were purchased from Extrasynthese (Genay-France). Acarbose was purchased from Serva (Heidelberg, Germany).

### 2.2. Plant Materials

*Lycium barbarum* L. (red goji) berries were obtained from a small farm in Reggio Calabria province (Calabria, Italy) in September 2022. The fully ripened goji berries (0.5 kg for each plant) were selected from trees cultivated in the same area. After harvesting, the berries were washed with distilled water and ground using a commercial blender (Power blend 9 JB 9040—Braun, Milan, Italy) to obtain a puree (50 mL).

### 2.3. Bread Preparation

Ciabatta bread is a typical Italian bread characterised by a crunchy external crust and a soft, very alveolar inside crumb. Ciabatta dough is characterised by high hydration, which involves the initial preparation of the “biga” formulated with Type 0 flour (300 W, 13 g of protein), dry brewer’s yeast, and water, and subsequently, after a first phase leavening, all the remaining ingredients are added in the “refreshment” phase.

**Figure 1:**
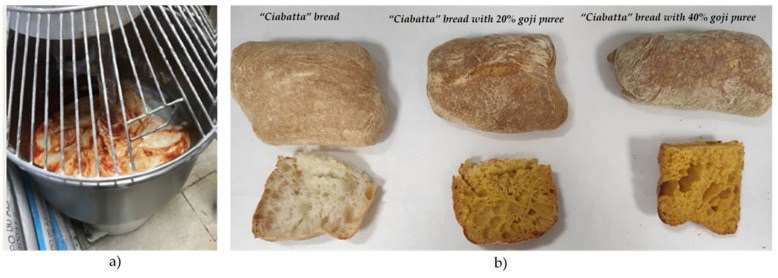
(**a**) Addition of goji fresh flesh puree to dough (**b**) from left to right ciabatta bread and enriched ciabatta bread (with 20% and 40% goji puree).

Finally, some folds are made, and the dough is divided into pieces of approximately 135 g/piece (15 × 9 cm^2^ and 3.8 cm deep) and baked at 230 °C for 15 min. The addition of the puree (% humidity: 78.38; ° Brix: 21.5) (Figure 1 and Figure S1) to the dough at different concentrations was carried out considering the moisture content of the goji puree and subtracting from this from the water content required by the traditional recipe, as reported in Table 1. Three pieces of approximately 400 g/piece, sufficient to obtain three loaves, which were placed in baking pans (10.5 × 6 cm^2^ and 6.5 cm deep).

### 2.4. Determination of a_w,_ Moisture Content, and Colourimetric Analysis

The moisture content of the bread and enriched bread was evaluated using the gravimetric method at 105 °C until constant weight was achieved with a Sartorius Moisture Analyser MA37. The results were expressed as percentage of moisture (%) [17]. Water activity (a_w_) was measured using an Aqualab LITE hygrometer (Decagon Devices Inc., Washington USA) [18]. CIELab chromatic parameters were evaluated using a Minolta CR 300 tristimulus colourimeter [19].

For each baking trial, the colour differences in crust and crumb were evaluated in terms of lightness (L*), red versus green (a*), and yellow versus blue (b*). In addition, chroma index (I) and hue angle (H) (II) were calculated to evaluate the degree of saturation/fullness and the amounts of redness and yellowness, respectively.

C* = (a2 + b2)1/2 (1)

H = arctan (b*/a*) (2)

The overall change in colour, ΔE*ab, between the control and samples G20 and G40 was calculated according to Mikulec et al. [16] following Equation (3):

(3) $$\Delta E*\mathrm{ab}=\sqrt{{\left(L_{c}^{*}-L_{g}{}^{*}\right)}^{2}+{\left(a_{c}^{*}-a_{g}{}^{*}\right)}^{2}{\left(b_{c}^{*}-b_{g}{}^{*}\right)}^{2}}$$

where a*_g,_ L*_g_, and b*_g_ are the colour parameters referring to G20 and G40 samples; a*_c_ referred to the control sample. The colour parameters enabled the calculation of the browning index, as follows:

$$\mathrm{BI}=\frac{100\;\left(x-031\right)}{0.17}$$

$$x=\frac{a_{g}^{*}+1.75L_{g}^{*}}{5.645L_{g}^{*}+a_{c-}^{*}3.012b_{g}^{*}}$$

### 2.5. Texture and Sensory Analyses

Texture analysis on samples was carried out by means of the cut slice test and texture profile analysis (TPA) using a TA-XT Plus Texture Analyser (Stable Micro Systems Ltd., UK). The data obtained were acquired and integrated using Exponent software 6.1.4.0 (Stable Micro Systems Ltd., UK).

The cut slice test was performed using a Blade Set with Knife probe (HDP/BSK set probe, Stable Micro Systems Ltd., UK) with testing parameters of 1 mm/s pre-test speed, 2 mm/s test speed, and 10 mm/s post-test speed; 100 mm test distance; 3 g trigger force, and 400 points/s acquisition rate. All tests were performed in triplicate.

The texture profile analysis was performed using a 100 mm cylindrical probe (P/100 compression platen probe, Stable Micro Systems Ltd., UK) on whole bread samples with testing parameters of 1 mm/s pre-test speed, 5 mm/s test speed, and 5 mm/s post-test speed; 20 mm test distance; 5 g trigger force, and 400 points/s acquisition rate. All tests were performed in triplicate. Hardness, cohesiveness, springiness, gumminess, resilience, and chewiness parameters were considered. The results were expressed as the mean value and statistically analysed using one-way ANOVA and post-hoc Tukey’s test multiple range test with IBM SPSS Statistics v.20 software (IBM Corp., Armonk, NY, USA).

Sensory analysis was carried out by quantitative descriptive analysis (QDA) with a trained panel of 8 adults recruited among faculty staff, composed of 5 males and 3 females, aged between 23 and 65 years. Panel members assessed samples following ISO 8586:2012 training for expert sensory assessors’ selection and training. QDA analysis was performed in sensory booths, presented in random order, served randomly at room temperature, and with cleansing between samples with natural mineral water. Tests were performed in triplicate and data was averaged. A reference bread sample was used to increase panel training on specific appearance and taste descriptors. Panelists’ QDA results were expressed as the mean of a 10-point structured scale ranging from 0 (absence) to 9 (extremely high) for visual, olfactory, taste, and texture descriptors (Table S1).

### 2.6. Extraction Procedure

Five grams of gojj fresh puree, goji puree after cooking treatment, flour, bread, and enriched bread (with 20% and 40% goji fresh flesh puree) were extracted using ultrasound-assisted extraction in hydroalcoholic ethanol solution (EtOH/H_2_O 80:20) in a 1:5 ratio (sample:solvent) (3 cycles, ultrasonic frequency of 40 kHz) in a water bath (3800-CPXH; Branson, Milan, Italy) at 28 °C for 45 min.

The goji puree was separated by centrifugation using a Nǜve NF 1200R (Saracalar Kümeevleri, Ankara, Turkey) (15 min at 5000 rpm). Before analysis, the sample was filtered through a 0.45 mm Millipore filter (GMF Whatman, Carlo Erba, Milan, Italy).

### 2.7. Total Phytochemical Content

Levels of some phytochemicals were analysed. In particular, the Total Phenol Content (TPC), Total Flavonoid Content (TFC), Total Anthocyanin Content (TAC), and Total Carotenoid Content (TCC) were the object of our study. TPC was evaluated as previously described [20]. Briefly, an extract was mixed with Folin-Ciocalteu reagent and 20% Na_2_CO_3_ solution. After 2 h, the absorbance was read using a UV-Vis Agilent 8453 spectrophotometer (Agilent Technologies, Milan, Italy). The results were expressed as mg gallic acid equivalents (GAE)/100 g fresh weight (FW). TFC was determined following flavonoid-aluminium complex methodology [21]. The extract was mixed with 2% aluminium chloride solution (25 °C, 15 min). The TFC was expressed as mg quercetin equivalents (QE)/100 g FW. For the evaluation of TAC, the differential pH method was applied, as previously described [22]. The results were expressed as equivalent mg of cyanidine-3-*O*-glucoside/100 g FW. The spectrophotometric analysis to analyse the TCC was carried out following the methodology reported by Fish et al. [23]. The results were expressed as equivalent mg β-carotene/100 g FW.

### 2.8. Goji Puree Selected Marker Quantification

Selected markers, such as caffeic acid, chlorogenic acid, *p*-coumaric acid, ferulic acid, 4-hydroxybenzoic acid, protocatechuic acid, and rutin, were quantified in fresh and processed goji puree extract using ultra-high-performance liquid chromatography (UHPLC) system. For this purpose, goji extract was analysed using a PLATINblue UHPLC (Knauer, Berlin, Germany), equipped with a binary pump system, a Knauer Blue Orchid column C18A (1.8 μm, 100 mm × 2 mm), coupled with a PLATINblue photo diode array detector (Knauer, Berlin, Germany), and using a flow rate of 0.4 mL min. For the quantification of constituents, the method reported by Zhao and Shi [24] was applied.

### 2.9. Antioxidant Activity

The antioxidant activity was evaluated using 2,2-azino-bis(3-ethylbenzothiazoline-6-sulfonic acid) (ABTS), 1,1-diphenyl-2-picrylhydrazyl (DPPH) radical scavenging activity, ferric reducing ability power (FRAP), and β-carotene bleaching tests, as previously detailed by Leporini et al. [25]. Briefly, in the FRAP test, FRAP reagent, acetate buffer (pH 3.6), FeCl_3_, HCl, and tripyridyltriazine were mixed and added to the extract at a concentration of 2.5 mg/mL. Butylated hydroxytoluene was used as a positive control. The ability of goji puree and derived products in the extract to protect a lipid substrate from peroxidation was assessed using the β-carotene bleaching test. Briefly, β-carotene solution, linoleic acid, 100% Tween 20, and extract at concentrations in the range 2.5–100 μg/mL were mixed. After incubation for 30 and 60 min at 25 °C, the absorbance was measured against a blank at t = 0. Propyl gallate was used as a positive control. In the ABTS test, a solution of ABTS radical cation was prepared and, after 12 h, was diluted with ethanol to reach an absorbance of 0.70 at 734 nm. The stabilised ABTS^+^ solution was added to extracts (1–400 μg/mL) and left to incubate for 6 min at 25 °C, and then the absorbance was read. In the DPPH radicals scavenging test, a solution of DPPH was mixed with extract (1–1000 μg/mL). After incubation for 30 min at room temperature, the absorbance was read. Ascorbic acid was employed as the positive control in both the ABTS and DPPH assays.

### 2.10. Inhibition of Pancreatic Lipase

To investigate potential lipase inhibition, a 96-well plate protocol was adopted [25]. Briefly, extract at concentrations from 2.5 to 40 mg/mL was mixed with the enzyme, 4-nitrophenyl octanoate, and Tris-HCl buffer (pH 8.5) and left to react for 30 min at 37 °C. Orlistat was used as a positive control.

### 2.11. Carbohydrate-Hydrolysing Enzymes Inhibitory Activity

The α-amylase and α-glucosidase inhibitory activities were assessed as detailed by Leporini et al. [25]. Briefly, in the α-amylase inhibitory assay, a starch solution was added to the extract at different concentrations (25 to 1000 μg/mL) and left to react with the enzyme (EC 3.2.1.1) at 25 °C for 5 min. In the α-glucosidase inhibitory activity test, a maltose solution was mixed with enzyme (EC 3.2.1.20), *o*-dianisidine solution, and peroxidase/glucose oxidase system-colour reagent. Then, the extract at different concentrations (25 to 1000 μg/mL) was added and left to react for 30 min at 37 °C. In both tests, acarbose was used as the positive control.

### 2.12. Statistical Analysis

The results were expressed as the mean of 3 different experiments ± standard deviation (SD). The concentration-response curve and the inhibitory concentration 50% (IC_50_) was calculated using GraphPad Prism version 4.0 for Windows software (GraphPad Software; San Diego, CA, USA). One-way analysis of variance (ANOVA) was performed with SPSS 17.0 software (SPSS Inc., Chicago, IL, USA). Significant differences were calculated according to Tukey’s multiple range tests. Differences at ** *p <* 0.01 were considered statistically significant.

## 3. Results and Discussion

Herein, we report the phytochemical content and potential bioactivity of goji puree, processed goji puree, flour, bread, and bread enriched with 20% and 40% goji puree. Samples were screened for their antioxidant properties and inhibitory activity of enzymes linked to metabolic syndrome.

### 3.1. Physico-Chemical and Colourimetric Parameters

**Figure 2:**
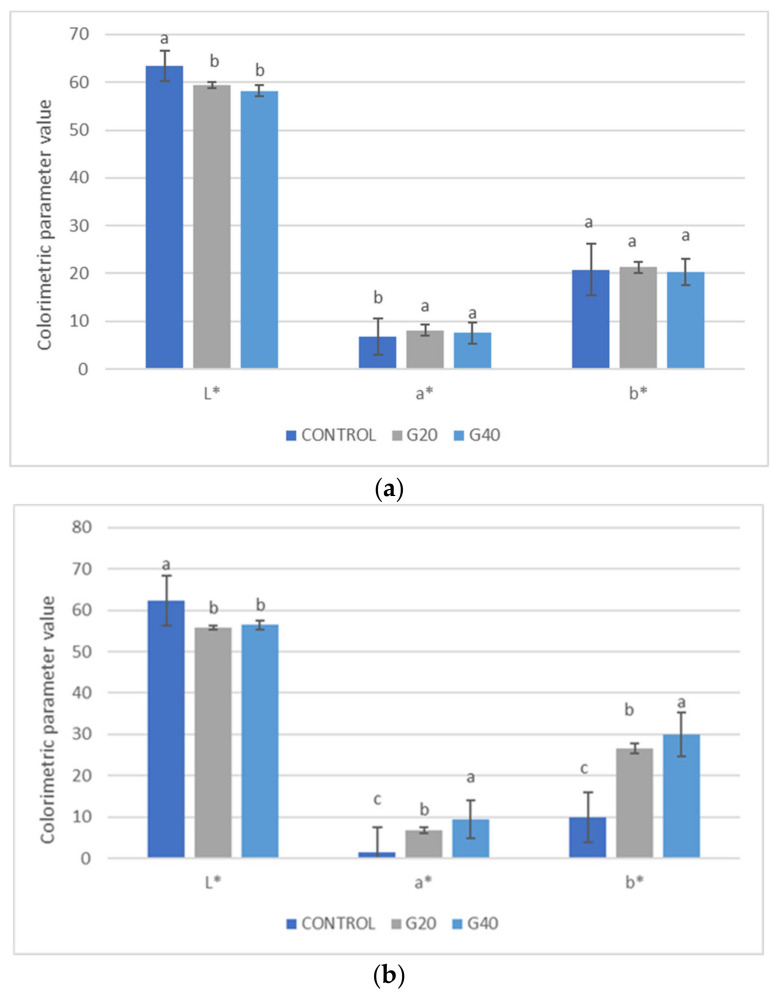
Colourimetric parameters (L*, a* and b*) in external (**a**) and internal (**b**) ciabatta bread and enriched ciabatta bread (with 20% and 40% goji puree). Differences between samples were evaluated by one-way ANOVA followed by Tukey’s test. Results followed by different letters are significantly different at ** *p* < 0.01.

The a_w_ parameter is a crucial variable in evaluating the stability of food since it is responsible for optimising the microbiological and physical properties of the food matrix, thus affecting the texture, flavour, odour, and colour [26]. a_w_ values of 0.91 and 0.90 were found for samples G20 and G40, respectively. These values were quite similar for the ciabatta bread used as the control (0.85) (*p* > 0.01). The moisture values were statistically different between the control bread and the samples enriched with goji puree, with values of 23.75% and 29.93% for C and G20, respectively (*p* > 0.01). As expected, the addition of different concentrations of goji fresh flesh puree to ciabatta bread resulted in a reduction in the lightness (L*) parameter measured on the bread crust (external portion), although no difference was recorded between samples G20 and G40 (Figure 2).

**Figure 3:**
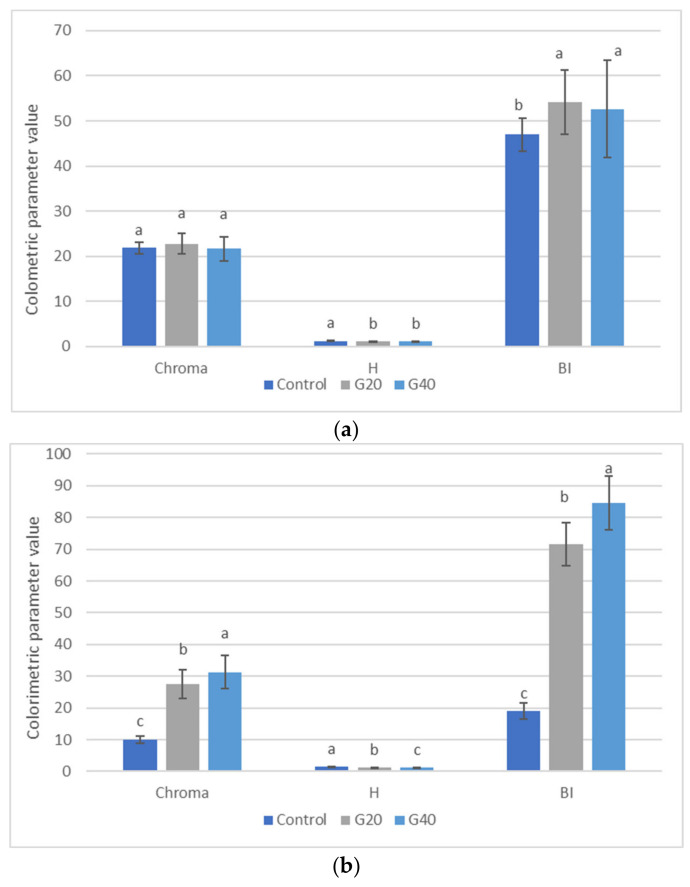
Chroma, hue angle, and browning index (BI) in external (**a**) and internal (**b**) ciabatta bread and enriched ciabatta bread (with 20% and 40% goji puree). Differences between samples were evaluated by one-way ANOVA followed by Tukey’s test. Results followed by different letters are significantly different at ** *p* < 0.01.

At the same time, as expected, the addition of goji resulted in an increase in the a* parameter (red-green). The same trend in L* and a* values was observed in the crumb and in this case, the increase in the a* parameter was proportional to the percentage of added goji fresh flesh puree (Figure 3). The reduction in bread lightness was also probably linked to the increase in moisture content following the addition of fruit pulp. Statistically significant differences were also recorded in the b* (yellow-blue) parameter, reaching a maximum value of 29.88 in sample G40.

These results suggested that the increased goji content, characterised by a high reducing sugar content, produced a darker product, which was probably linked to the Maillard and caramelisation reactions [27,28].

Interestingly, the hue angle and chroma parameters (Figure 3) highlighted that the effect of goji addition was more evident on the crumb. Particularly, the higher percentage of fruit pulp resulted in a higher chroma value. Only slight variations were observed for the hue angle of both crust and crumb.

As also found by Balestra et al. [29], who studied the effect of powdered ginger on bread, the addition of goji probably determined the chromatic variation toward a darker colour. Consequently, the crumb assumed a brighter colour than that of the control bread. Increasing the amount of goji puree resulted in an increase in total colour difference (data not shown) and the browning index. In terms of crumb colour change, a ΔE value of 24.11 was calculated between the control and sample G40, while a lower ΔE value was obtained between the control and sample G20 (20.71). In contrast, a lower colour change was recorded for the bread crust, with ΔE values of 5.80 and 7.59 for samples G20 and G40, respectively. The colour changes could be due to the original colour of the raw materials used, which could determine a darker brown colour in the final baked product. Product formulation is also a critical factor for browning development. The accumulation of coloured compounds during baking is influenced by different factors, such as sugar content, water activity, and processing temperature [30]. The results of the browning index indicated that the effect of addition was more pronounced in the breadcrumb for the higher concentration of goji. In contrast, the effect of coloured compounds was less pronounced in the bread crust, showing a higher value for sample G20.

### 3.2. Texture and Sensory Results

Rheological parameters and TPA are widely applied to determine food texture. The cut slice test gives information about basic structural aspects of bread. With regard to texture profile analysis, the key parameters considered for bread acceptance are hardness, cohesiveness, and chewiness (intended as the main descriptive parameters to quantify the energy required to cut and break a semi-solid food until it is ready to swallow), springiness (the rate at which a deformed sample reforms itself), and gumminess and resilience, (measurements of sample recovery behaviour from deformation in relation to speed and force derived) [31]. The cut slice test performance of the bread is reported in Table 2. Goji-modified bread (samples G20 and G40) demonstrated considerably lower hardness values than the control (*p* < 0.01).

Table 3 shows the TPA results of the control and modified breads. The TPA hardness parameter, in opposition to results of the cut slice test, did not show significant differences among bread samples. This could be ascribed to the different instrument fixtures used for testing.

A non-significant difference was observed for springiness among the bread samples. Instead, the modified bread differentiated from the control in other parameters, such as cohesiveness, gumminess, chewiness, and resilience, while demonstrating similar behaviour for both percentages of goji supplementation.

**Figure 4:**
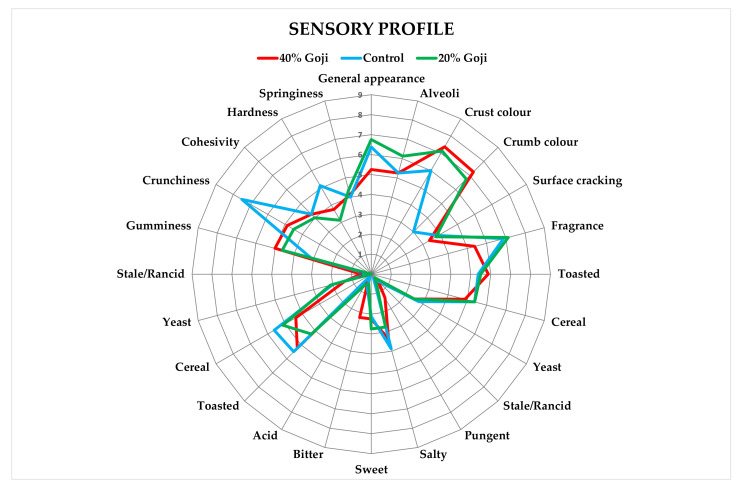
Comparison between control ciabatta and ciabatta enriched with goji puree.

The sensory profiles for the control and modified breads are shown in Figure 4. The presence of crust and crumb colouring were found as the main appearance descriptors while fragrance-flavour was the main olfactory descriptor. Moreover, the principal taste descriptors were toasted and cereal. All textural descriptors were recognised by panellists, with particular emphasis on crunchiness. The enriched breads with both 20% and 40% goji fresh flesh puree differentiated from the control bread with significantly higher appearance descriptors. Taste and flavour descriptors did not show significant differences among samples (*p* < 0.01).

Among the textural descriptors, crunchiness was significantly decreased in the enriched breads compared to the control.

Previously, Ziemichód et al. [32] formulated a goji-enriched gluten-free bread. For the development of this product, rice and maize flour (50:50) were used. Dried goji berries were added in percentages from 3% to 15%. The addition of dried goji did not influence the volume of the bread until reaching 12%. At the same time, goji addition contributed to an increase in the redness of the breadcrumb and a reduction in the L* parameter, which agreed with our results. The addition of goji, even at high percentages, reduced the hardness of the breadcrumbs, and at the same time, resulted in a statistically significant increase in bread elasticity. An increase in the cohesion of the crumb was observed with the addition of goji berries from 3% to 6%. Moreover, goji addition to the gluten-free bread had a positive effect on its aroma and taste.

### 3.3. Total Phytochemical Content and Selected Marker Quantification

Several bioactive compounds were quantified in our samples. Among them. phenolic compounds play a prominent role due their biological activities [33]. In general, bread enriched with goji maintained good levels of TPC and TFC compared to berry pulp alone, despite cooking, with TPC values of 49.67 and 54.88 mg gallic acid equivalent (GAE)/100 g fresh weight (FW) for bread enriched with 20% (G20) and 40% (G40) goji fresh fruit flesh, respectively. The TFC values were 40.04 and 43.49 mg quercetin equivalent (QE)/100 g FW for samples G20 and G40, respectively (Table 4).

A similar trend was also observed for TAC, where fresh flesh puree had a TAC value of 0.03 mg equivalent of cyanidine-3-*O*-glucoside/g of FW. This value was halved after cooking at a temperature of 230 °C for 15 min. The TCC values ranged from 0.04 to 0.15 mg β-carotene/100 g FW. A slightly higher TPC value was reported by Donno et al. [34] in dried goji berries collected in Northern Italy (from 255.87 to 281.91 mg GAE/100 g FW).

Previously, Benchennouf et al. [35] studied the impact of extraction with different solvents for dried goji berries from Greece and found great variability in TPC values depending on the solvent, with values of 14.13 and 109.72 mg GAE/dry extract for water and ethyl acetate, respectively. Lower TPC values were obtained for dried goji berries from Turkey extracted with methanol (9.04 mg GAE/ dry extract) [36]. Values from 7.26 to 9.01 mg GAE/dry extract and from 9.77 to 12.32 mg CAE/dry extract were found for dried Chinese *L. barbarum* berry extracts [37]. In the same sample, TAC values from 60.52 to 82.58 mg CAE/g extract were found. For TCC, an average value of 197.8 mg/100 g was found, whereas Italian berries had double the value of TCC (355 mg/100 g). These results evidenced that Italian goji berries have higher biological and nutritional value than those of Asian origin [38].

Freeze-dried goji samples were analysed by UHPLC to quantify selected markers (Table 5). As expected, a reduction in their content was observed after cooking at 230 °C, although the results were variable. Among the identified compounds, protocatechuic acid was the most abundant with a value of 48.84 μg/g freeze-dried sample, followed by rutin with 43.73 μg/g freeze-dried.

An appreciable amount of chlorogenic acid was detected in the fresh goji sample (41.57 μg/g freeze-dried). However, its content was halved after simulating the cooking conditions.

The growing environment, harvesting time, and storage conditions affect the phenolic composition of goji fruits [39]. As reported in Table 5, goji puree was characterised by high contents of phenolic acids and the flavonoid rutin. The obtained results were consisted with the literature data. Pedro et al. [40] identified chlorogenic, 4-hydroxybenzoic, caffeic, and *p*-coumaric acids as the main abundant phenolic acids, while the flavonoid rutin was the most abundant with a value of 665 mg/100 g extract. Additionally, Duan al. [41] reported that the phenolic composition of goji fruit was characterised by high content of caffeic, chlorogenic, and ferulic acids as well as rutin. According to Maghsoudlou et al. [42], a loss of bioactive compounds was observed in processed goji.

However, it is interesting to note that when rutin, considered by the European Pharmacopeia as a quality marker of goji berries [43], was measured by UHPLC in ciabatta bread and goji-enriched bread, it was evident that the flavonoid was absent in the control bread and its value increased in proportion to the percentage of goji added to the enriched bread, reaching 19.79 μg/mL in sample G40 (data not shown). This flavonoid is able to exert antioxidant effects and inhibitory activities against carbohydrate-hydrolysing enzymes and lipase [44,45,46]. Preservation of rutin during baking is an interesting possibility to enrich bread with antioxidant effects, as evidenced for other types of natural compounds [47].

### 3.4. Antioxidant Activity

The antioxidant properties of flour, goji fresh flesh puree, processed goji flesh puree, ciabatta bread, and goji-functionalised ciabatta bread extracts were assessed using different in vitro methods, namely the FRAP, β-carotene bleaching, ABTS, and DPPH tests (Table 6).

Goji flesh puree was characterised by the highest FRAP value of 62.26 μM Fe(II)/g. This value was comparable to the positive control BHT (FRAP value 63.26 μM Fe(II)/g). Simulation of the cooking process on goji flesh puree reduced the FRAP value to 47.47 μM Fe(II)/g (sample GS).

Goji-enriched ciabatta bread retained good ferric reducing ability power, with a FRAP value of 55.81 μM Fe(II)/g for sample G40. All samples exhibited a promising ability to protect against lipid peroxidation. In particular, IC_50_ values of 6.88 and 6.52 μg/mL were recorded for samples G20 and G40 after incubation for 30 min, respectively. No significant differences were noted between G and GS activity, with IC_50_ values of 7.12 and 7.53 μg/mL, respectively.

Moderate DPPH radical scavenging activity was observed, with IC_50_ values in the range of 66.82–160.36 μg/mL for G and F, respectively. Although the thermal process decreased the phenolic compound content, higher DPPH values were recorded for processed goji fruit. This could be explained by considering the formation of intermediate products derived from the Maillard reaction with significant antioxidant proprieties [42].

A different behaviour was found using the ABTS radical cation. In fact, in this test, all samples were characterised by high activity, except flour. Enriched ciabatta bread with 20% and 40% goji fresh flesh puree exhibited IC_50_ values of 27.26 and 26.12 μg/mL, respectively.

Several studies demonstrated that goji berries have less antioxidant potential than blueberries or blackcurrants, but at the same time, they have more than raspberries or oranges [34]. *Lycium ruthenicum* (black goji) berries have more potent antioxidant activity than red goji berries [37,48]. Dried goji berry fruits from an *L. barbarum* cultivar cultivated in Greece showed good free radical scavenging activity, with IC_50_ values in the range 1.29–3.00 mg/mL for DPPH and 0.39–1.10 mg/mL for ABTS, respectively [49]. Mocan et al. [43] reported the DPPH radical scavenging activity of goji berries from different origins and found activities ranging from not active to 40.32 mg TE/g DW extract (for the Italian sample), whereas Zhang et al. [50] found DPPH values in the range of 35.88–85.46 μM TE/g FW in different Chinese goji genotypes. A remarkable variability was detected in the ferric reducing ability power, with FRAP values ranging from 15.71 to 51.93 mg TE/g DW extract [43]. Previously, Ruffo et al. [51] showed the higher antioxidant potential of Calabrian goji extract in comparison to the Chinese variety.

### 3.5. Inhibition of Enzymes Linked to Type 2 Diabetes and Obesity

The growing consumer interest in functional foods capable of preventing obesity and related diseases, such as type 2 diabetes, is a topic of great interest for researchers in the food science and technology sector. To our knowledge, no previous studies have investigated the ability of bakery products enriched with goji to counteract enzymes linked to diabetes and obesity. All investigated samples exerted enzyme inhibitory activities in a concentration-dependent manner (Table 7). Goji fresh flesh puree exerted the highest activity, with an IC_50_ value of 48.02 μg/mL, followed by processed flesh puree extract (IC_50_ of 86.46 μg/mL).

Enriched ciabatta bread evidenced a lipase inhibitory activity, with IC_50_ values of 120.44 and 153.97 μg/mL for samples G40 and G20, respectively.

Moderate α-glucosidase inhibitory activity was observed for sample G, with an IC_50_ value of 68.02 μg/mL, whereas values of 146.51 and 126.93 μg/mL were found for enriched ciabatta bread with 20% and 40% goji fresh flesh puree, respectively.

An IC_50_ values of 107.23 and 399.31 μg/mL were recorded against α-amylase for fresh flesh puree and flour, respectively.

Recently, Wojdyło et al. [52] reported the inhibitory activity against carbohydrate-hydrolysing enzymes of 21 cultivars of *L. barbarum*, finding that α-amylase inhibitory activity ranged from 9.6% to 82.6% whereas values from 5.7% to 15.3% were recorded against α-glucosidase. Moreover, Zou et al. [53] demonstrated the hypoglycaemic potential of goji berries by promoting the translocation of the glucose transporter, GLUT4, in skeletal muscle.

At the same time, Cai et al. [54] reported that the administration of goji liposaccharides fraction (300 mg/day) for 3 months reduced glucose and insulinogenic indexes and increased the HDL value. High α-glucosidase and α-amylase inhibitory activities were found for *L. chinense* (‘Big Lifeberry’), with IC₅₀ values of 9.9 and 33.4 mg/mL, respectively [55]. Moreover, it was reported that lipopolysaccharides can notably inhibit the activities of α-amylase and lipase [56]. A comparison between wild and cultivated goji leaves showed that goji could counteract carbohydrate-hydrolysing enzymes, with higher inhibitory activity against α-glucosidase than against α-amylase [57].

## 4. Conclusions

Recent years have witnessed the evolution of consumer preferences as they become increasingly aware of the importance of a diet aimed at improving their quality of life and preventing chronic degenerative diseases. Numerous bakery products enriched in functional ingredients have been formulated, including prebiotics and antioxidant compounds, to obtain a competitive advantage in the market compared to traditional products. In this context, this work studied, for the first time, the enrichment of a traditional Italian bread, known as “ciabatta”, with different percentages of goji fresh flesh puree.

Consumption of this functional bread, with a strong relationship with the territory, could help prevent metabolic syndrome and associated pathologies due to its antioxidant properties and inhibitory activities against key enzymes, such as α-amylase, α-glucosidase, and pancreatic lipase. The enriched bread maintained good levels of bioactive phytochemicals and antioxidant effects. Moderate inhibitory activities against α-amylase, α-glucosidase, and pancreatic lipase were found. The lack of correlation between a single class of phytochemicals and bioactivity supports the synergism of goji phytochemicals.

From a sensory point of view, the addition of fresh goji pulp puree maintained the appearance of the bread while it did not change the characteristics related to flavour and taste.

Collectively, our results demonstrate the possibility of transforming traditional ciabatta bread into a new functional bakery product to be consumed daily.

## Figures and Tables

**Table 1:** Bread Formulation.

C^	G20	G40
Chariot		
1350 g flour type 0	1350 g flour type 0	1350 g flour type 0
607 g water	607 g water	607 g water
6.75 g *Saccharomyces cerevisiae*	6.75 g *Saccharomyces cerevisiae*	6.75 g *Saccharomyces cerevisiae*
Refreshments		
150 g flour type 0	150 g flour type 0	150 g flour type 0
6.75 g malt	6.75 g malt	6.75 g malt
547 g water	317 g water *	131 g water *
37.5 g salt	37.5 g salt	37.5 g salt
	230 g goji fresh flesh puree	416 g goji fresh flesh puree

^^^ Quantities referring to 1.5 kg of dough. * Water content was modified according to the concentration of goji puree added. C: Bread; G20: Bread enriched with 20% goji fresh flesh puree; G40: Bread enriched with 40% goji fresh flesh puree.

**Table 2:** Rheological properties of goji enriched bread.

Bread Sample	Cut Slice Test
	Hardness (g)
C	13,945.62 ± 1263.08 ^a^
G20	9965.30 ± 923.77 ^b^
G40	9113.69 ± 1958.85 ^b^
**Sign.**	**

C: bread; G20: bread enriched with 20% goji puree; G40: bread enriched with 40% goji puree. Data are reported as mean ± standard deviation (SD) (*n* = 3). Differences within and between groups were evaluated by one-way ANOVA followed by Tukey’s multiple range test. Results followed by different letters in a same column are significantly different at ** *p* < 0.01.

**Table 3:** Texture profile analysis (TPA) of rheological characteristics of bread.

Sample	Texture Profile Analysis (TPA) Test					
	Hardness (g)	Springiness (mm)	Cohesiveness	Gumminess (g)	Chewiness (g)	Resilience
Control	6871.96 ± 1467.12 ^a^	0.833 ± 0.031 ^a^	0.525 ± 0.077 ^a^	3550.68 ± 521.63 ^a^	2942.90 ± 468.30 ^a^	0.199 ± 0.042 ^a^
G20	6556.93 ± 3925.43 ^a^	0.871 ± 0.017 ^a^	0.676 ± 0.016 ^b^	4412.09 ± 2574.79 ^b^	3847.27 ± 2258.49 ^b^	0.310 ± 0.014 ^b^
G40	7616.37 ± 3824.32 ^a^	0.868 ± 0.022 ^a^	0.672 ± 0.030 ^b^	5097.56 ± 2485.90 ^b^	4449.18 ± 2245.96 ^b^	0.316 ± 0.036 ^b^
**Sign.**	ns	ns	**	**	**	**

C: bread; G20: bread enriched with 20% goji puree; G40: bread enriched with 40% goji puree. Data are reported as mean ± standard deviation (SD) (*n* = 3). Differences within and between groups were evaluated by one-way ANOVA followed by Tukey’s multiple range test. Results followed by different letters in a same column are significantly different at ** *p* < 0.01. ns: not significant.

**Table 4:** Phytochemical content of flour, goji fresh flesh puree, processed goji flesh puree, ciabatta bread, and goji-functionalised ciabatta bread extracts.

Sample	TPC	TFC	TCC	TAC
F	44.82 ± 1.91 ^e^	38.72 ± 1.7 ^e^	ND	ND
G	189.1 ± 3.83 ^a^	93.22 ± 2.4 ^a^	0.15 ± 0.91 ^a^	0.03 ± 0.00 ^a^
GS	51.23 ± 2.02 ^d^	45.61 ± 1.9 ^d^	0.09 ± 0.20 ^b^	0.01 ± 0.00 ^b^
C	40.91 ± 1.54 ^f^	30.47 ± 1.8 ^f^	0.04 ± 0.01 ^d^	0.01 ± 0.00 ^b^
G20	49.67 ± 3.21 ^c^	40.04 ± 3.2 ^c^	0.08 ± 0.01 ^c^	0.01 ± 0.00 ^b^
G40	54.88 ± 3.36 ^b^	43.49 ± 3.30 ^b^	0.08 ± 0.01 ^c^	0.01 ± 0.00 ^b^
***Sign.***	**	**	**	ns

F: flour; G: fresh goji puree; GS: cooked goji puree; C: bread; G20: bread enriched with 20% goji puree; G40: bread enriched with 40% goji puree. TPC: mg GAE/100 g FW. TFC: mg QE/100 g FW. TCC: mg β-carotene/100 g FW. TAC: cyanidine-3-*O*-glucoside/100 g FW. ND: not detected. Data are reported as mean ± standard deviation (SD) (*n* = 3). Differences within and between groups were evaluated by one-way ANOVA followed by Tukey’s multiple range test. Results followed by different letters in the same column are significantly different at ** *p* < 0.01. ns: not significant.

**Table 5:** UHPLC analysis of selected compounds in freeze-dried goji samples (μg/g).

Compound	G	GS	Sign.
Protocatechuic acid	48.84 ± 0.42	5.17 ± 0.15	**
4-Hydroxybenzoic acid	27.70 ± 0.31	17.81 ± 0.32	**
Chlorogenic acid	41.57 ± 0.52	27.69 ± 0.12	**
Caffeic acid	11.33 ± 0.21	7.83 ± 0.09	**
*p*-Coumaric acid	6.84 ± 0.12	5.09 ± 0.11	**
Ferulic acid	14.77 ± 0.24	9.28 ± 0.12	**
Rutin	43.73 ± 0.52	17.83 ± 0.23	**

G: fresh goji puree; GS: cooked goji puree; Data are reported as mean ± standard deviation (SD) (*n* = 3). Differences within and between groups were evaluated by one-way ANOVA followed by Tukey’s test: ** *p* < 0.01.

**Table 6:** Antioxidant activity of fresh and processed goji puree and enriched ciabatta bread.

Sample	FRAP Test	β-Carotene Bleaching Test		DPPH Test	ABTS Test
		t = 30 min	t = 60 min		
	μM Fe(II)/g	IC_50_ (μg/mL)	IC_50_ (μg/mL)	IC_50_ (μg/mL)	IC_50_ (μg/mL)
F	7.18 ± 0.80 ^e^	13.53 ± 1.42 ^d^	46.03 ± 3.54 ^e^	160.36 ± 4.58 ^e^	105.48 ± 4.93 ^e^
G	62.26 ± 1.12 ^a^	7.12 ± 0.98 ^b^	13.83 ± 1.20 ^c^	66.82 ± 3.56 ^a^	13.15 ± 1.20 ^a^
GS	47.47 ± 0.70 ^c^	7.53 ± 0.45 ^b^	14.99 ± 1.24 ^c^	100.52 ± 7.92 ^d^	17.22 ± 1.25 ^b^
C	10.41 ± 1.02 ^d^	8.73 ± 1.09 ^c^	22.66 ± 2.89 ^d^	100.31 ± 3.76 ^d^	37.19 ± 0.87 ^d^
G20	46.94 ± 1.44 ^c^	6.88 ± 0.83 ^a^	12.53 ± 2.78 ^b^	98.80 ± 2.21 ^c^	27.26 ± 1.34 ^c^
G40	55.81 ± 1.27 ^b^	6.52 ± 0.53 ^a^	9.26 ± 0.87 ^a^	72.53 ± 7.89 ^b^	26.12 ± 1.45 ^c^
***Sign***.	**	**	**	**	**

IC_50_: inhibitory concentration 50% F: flour; G: fresh goji puree; GS: cooked goji puree; C: Bread; G20: bread enriched with 20% goji puree; G40: bread enriched with 40% goji puree. Data are reported as mean ± standard deviation (SD) (*n* = 3). Ascorbic acid, BHT and Propyl gallate were used as positive control in antioxidant tests: Ascorbic acid IC_50_ of 5.04 ± 0.81 μg/mL in DPPH test, and 1.72 ± 0.14 μg/mL in ABTS; BHT 63.26 ± 2.31 MFe (II)/g in FRAP test; Propyl gallate IC_50_ of 0.09 ± 0.04 μg/mL in β-carotene bleaching test at t = 30 min and 0.09 ± 0.04 μg/mL in β-carotene bleaching test at t = 60 min. Differences within and between groups were evaluated by one-way ANOVA followed by Tukey’s multiple range test. Results followed by different letters in a same column are significantly different at ** *p* < 0.01.

**Table 7:** Lipase, α-glucosidase, and α-amylase inhibitory activities [IC_50_ (μg/mL)] of fresh and processed goji fresh puree and enriched ciabatta bread.

Sample	Lipase	α-Glucosidase	α-Amylase
F	253.61 ± 3.67 ^f^	250.02 ± 4.35 ^f^	399.31 ± 4.68 ^f^
G	48.02 ± 1.69 ^a^	68.02 ± 1.21 ^a^	107.23 ± 1.30 ^a^
GS	86.46 ± 1.02 ^b^	109.06 ± 2.04 ^b^	156.74 ± 1.85 ^c^
C	246.17 ± 3.45 ^e^	196.92 ± 2.37 ^e^	336.71 ± 4.04 ^e^
G20	153.97 ± 3.78 ^d^	146.51 ± 2.01 ^c^	233.55 ± 2.10 ^d^
G40	120.44 ± 1.25 ^c^	126.93 ± 2.69 ^d^	171.76 ± 1.39 ^b^
***Sign***.	**	**	**

F: flour; G: fresh goji puree; GS: cooked goji puree; C: Bread; G20: bread enriched with 20% goji puree; G40: bread enriched with 40% goji puree. Data are reported as mean ± standard deviation (SD) (*n* = 3). Orlistat was used as a positive control in lipase test (IC_50_ value of 37.4 ± 1.0 μg/mL) whereas acarbose in α-glucosidase and α-amylase (IC_50_ values of 35.5 ± 1.10 and 50.12 ± 1.13 μg/mL, respectively). Differences within and between groups were evaluated by one-way ANOVA followed by Tukey’s multiple range test. Results followed by different letters in the same column are significantly different at ** *p* < 0.01.

## Data Availability

The data are contained within the article.

## Supplementary Materials

The following supporting information can be downloaded https://www.mdpi.com/article/10.3390/foods12030566/s1, Figure S1. Flowchart of ciabatta bread and enriched bread preparation; Table S1. Sensory descriptors list.
