# Supplementary material for: Ciabatta Bread Incorporating Goji (Lycium barbarum L.): A New Potential Functional Product with Impact on Human Health
Source: Foods. 2023 Jan 27;12(3):566. doi: 10.3390/foods12030566 (PMC9913991; doi:10.3390/foods12030566)
Supplement: Supplementary file 1 [file foods-12-00566-s001.zip › foods-2185561-supplementary.pdf]

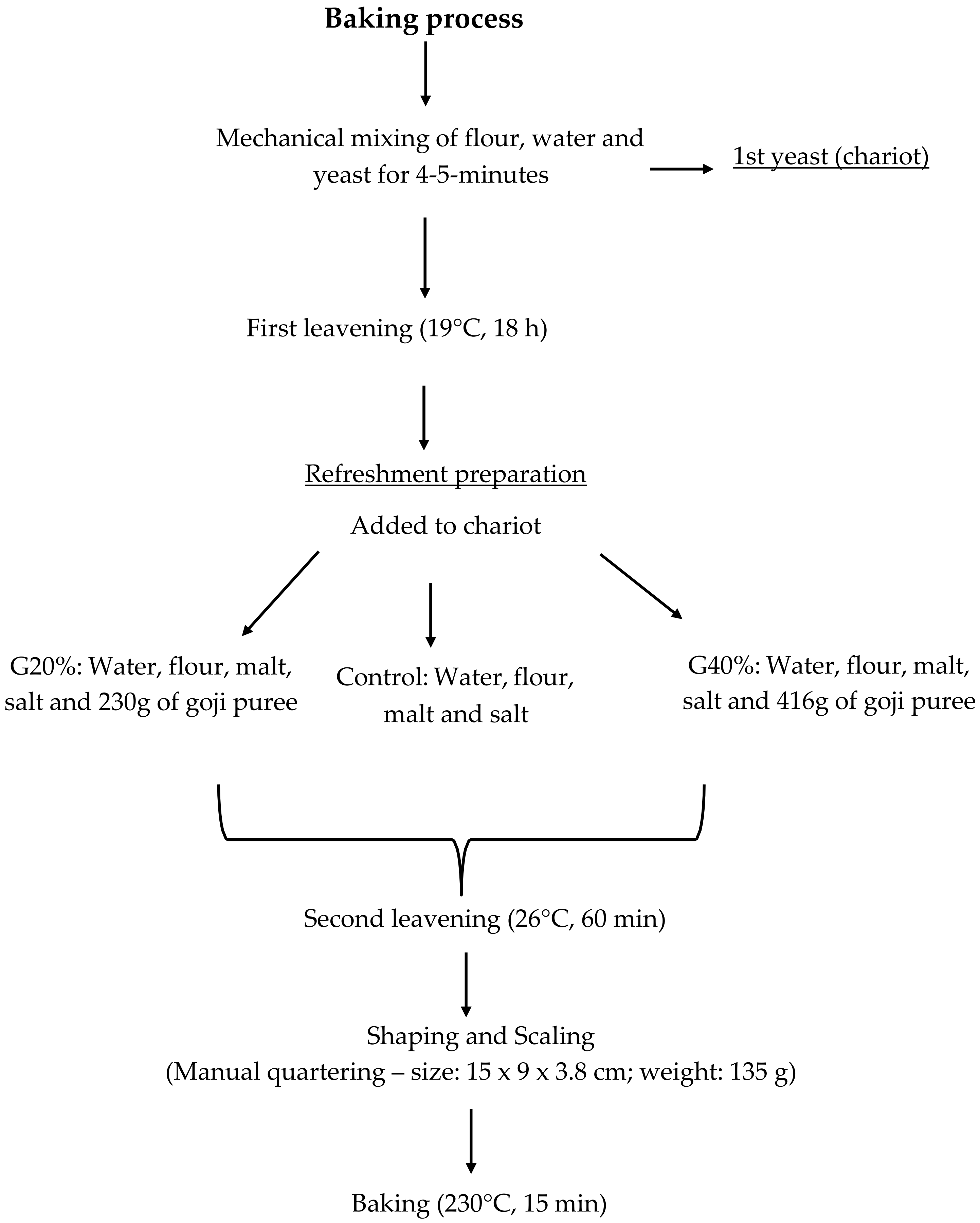

Figure S1. Flowchart of "Ciabatta" bread and enriched bread preparation

Table S1 - Sensory descriptors list

Category	Descriptor	Definition
**Appearance**	General appearance	
	Alveoli	Presence and intensity of alveoli in crumb
	Crust colour	
	Crumb colour	
	Surface cracking	
**Olfactory**	Fragrance	Product typical flavor intensity
	Toasted	
	Cereal	
	Yeast	
	Stale/Rancid	
	Pungent	
**Taste**	Salty	Product typical taste intensity
	Sweet	
	Bitter	
	Acid	
	Toasted	
	Cereal	
	Yeast	
	Stale/Rancid	
**Texture**	Gumminess	
	Crunchiness	
	Cohesivity	
	Hardness	
	Springiness	
